# Antibodies against Lewis antigens inhibit the binding of human norovirus GII.4 virus-like particles to saliva but not to intestinal Caco-2 cells

**DOI:** 10.1186/s12985-016-0538-y

**Published:** 2016-05-21

**Authors:** Noelia Carmona-Vicente, David J. Allen, Jesús Rodríguez-Díaz, Miren Iturriza-Gómara, Javier Buesa

**Affiliations:** Department of Microbiology, School of Medicine, University of Valencia, Avda. Blasco Ibáñez, 17, 46010 Valencia, Spain; Virus Reference Department, Public Health England, London, UK; CIMI, Institute of Infection and Global Health, University of Liverpool, Liverpool, UK; NIHR Health Protection Research Unit in Gastrointestinal Infections, University of Liverpool, Liverpool, UK

**Keywords:** Human norovirus (NoV), Virus-like particles (VLPs), Caco-2 cells, GII.4 genotype, Histo-blood group antigens (HBGAs), Receptor binding

## Abstract

**Background:**

Human noroviruses (NoVs) are the main cause of gastroenteritis worldwide. The most commonly detected NoV strains belong to the genetically diverse GII.4 genotype, with new pandemic variants emerging periodically. Despite extensive efforts, NoV investigation has been hampered by the lack of an effective in vitro cell culture system. However, NoV-derived recombinant virus-like particles (VLPs) resembling empty capsids are good surrogates for analysing NoV antigenicity and virus-ligand interactions. NoV VLPs have been reported to bind to histo-blood group antigens (HBGAs). We have analysed the ability of NoV VLPs derived from GI.1 genotype and from three GII.4 genotype variants, GII.4-1999, GII.4-2004 and GII.4-2006b, to bind to porcine gastric mucin (PGM), human saliva and differentiated human intestinal Caco-2 cells (D-Caco-2 cells).

**Results:**

Distinct patterns of saliva binding with the NoV GII.4 variant VLPs were observed, although they bound to D-Caco-2 cells independently of the expression of HBGAs. Monoclonal antibodies against Lewis antigens were able to block the binding of NoV VLPs to saliva, but not to D-Caco-2 cells. Blocking HBGAs on the surface of D-Caco-2 cells with specific monoclonal antibodies did not affect NoV VLP binding to cellular membranes. Co-localisation of Lewis y (Le^y^) and H-type 2 antigens with NoV VLPs was not observed by immunofluorescence assays.

**Conclusion:**

Although the binding of NoV VLPs of GII.4 genotype variants to human saliva samples occur with distinct HBGA binding patterns and can be blocked by antibodies against Lewis antigens, their attachment to D-Caco-2 cells can be mediated by other receptors, which still need further investigation.

## Background

Noroviruses (NoVs) are the main cause of sporadic cases and outbreaks of acute gastroenteritis and are associated with a large burden of disease globally [1–3]. NoVs are small, non-enveloped, icosahedral viruses with a positive single-stranded RNA genome; they belong to the *Caliciviridae* family and are genetically classified into 6 genogroups (GI-GVI) with a recently proposed genogroup VII [4], although genogroup I (GI) and GII cause most human NoV infections. Despite this diversity over the past two decades most reported NoV outbreaks and epidemics have been caused by NoV GII.4 genotype. Phylogenetic analyses of the GII.4 strains circulating in the last 20 years have shown that this genotype can be divided into distinct variants, which peak and wane over time in a similar pattern to that described for influenza viruses [5–7]. Several studies have linked NoV susceptibility to histo-blood group antigens (HBGAs), namely with the secretor status associated with the presence of at least one functional *FUT2* allele, and with Lewis antigens (Le^a^ and Le^b^), determined by the *FUT3* gene [8, 9]. The HBGAs, including the ABO, secretor and Lewis families, are distributed on cell membranes and mucosal epithelia with high polymorphism. HBGAs are synthesized from various disaccharide precursors through sequential additions of monosaccharides with specific linkages catalysed by different glycosyltransferases [10]. The syntheses of the secretor, Lewis and ABO antigens are catalyzed by an α-1,2 fucosyltransferase (FUT2), an α-1,3 or α-1,4 fucosyltransferase (FUT3) and two glycosyltransferases (A and B enzymes), respectively. Homozygote carriers of inactive *FUT3* alleles essentially lack Le^a^ and Le^b^ antigens; such individuals are denoted Lewis-negative and constitute about 5 % of the Caucasian population. Secretor-positive individuals express Le^b^ antigen, while secretor-negative individuals express Le^a^ antigen [11]. Human NoVs are known to recognize HBGAs as attachment factors, with different NoV strains showing different properties regarding the ability to bind to different antigens [8, 10].

The NoV genome is organized in three open reading frames (ORFs). The VP1, encoded by ORF2, is the major capsid protein, which is further organized into the N-terminal (N), the shell (S), and the protruding (P) domains. The P domain is divided into two subdomains: P1 and P2 [12]. The P1 subdomain forms the anchoring portion of the P dimer connecting it to the S domain, while the P2 subdomain is exposed on the surface of the capsid protein and is the most variable region of the virus. The main epitopes for immunorecognition and the histo-blood group antigen (HBGA) binding domains reside within this P2 subdomain. The emergence and accumulation of mutations along the P2 subdomain is the main driver of evolution for GII.4 strains, which results in epidemic strains with altered antigenicity and HBGA binding properties [13–16]. It has been reported that NoVs attach to either HBGA expressed on the gastroduodenal epithelial cells of secretor-positive individuals [17, 18]. Human secretor positive saliva and synthetic HBGAs have been used in VLP binding and/or blocking assays in different studies [19–21]. However, it has also been shown that NoV can bind to enterocytes independently of HBGAs [22]. Human NoVs have for long time been elusive to propagation in cell cultures [23, 24], although it has been recently reported that human NoVs can infect B lymphocytes in the presence of HBGA-expressing bacteria [25]. Caco-2 cells, originally derived from a human colonic adenocarcinoma, show morphologic and physiologic markers of differentiation characteristic of the mature small intestine enterocytes, express carbohydrates of the histo-blood group family on their surface, and allow significant attachment of norovirus VLPs mainly when these cells are differentiated [26].

In this study we have assessed the binding properties of VLPs of different variants of NoV GII.4 genotype to Caco-2 cells. Moreover, blockade activity of the VLP binding by porcine gastric mucin (PGM) and monoclonal antibodies (mAbs) against NoV VLPs, anti-Lewis antigens (Le^a^, Le^b^, Le^x^ and Le^y^) or anti-H antigens (H1 and H2) mAbs was investigated in order to better understand the interactions between NoVs and the cellular surface of intestinal cells.

## Results

### Distribution of Lewis antigens, secretor status and ABO types among the saliva donors

Lewis antigens, secretor status (FUT2) and ABO blood group of the 22 saliva donors were determined to analyse the VLP binding patterns. By ABO typing, 10 subjects were type A, 5 type B, 7 type O and none of them were type AB. According to the secretor status, 86.4 % (19/22) were secretors, 22.7 % (5/22) were secretor homozygous (SeSe), 63.6 % (14/22) were secretor heterozygous (Sese), and the remaining 13.6 % (3/22), were non-secretors (sese). The anti-Lewis antigen mAb-based EIA classified the saliva samples into four groups according to Lewis antigens patterns: Le^a+ b+^/Le ^x- y+^ (77.3 %), Le^a+ b+^/Le ^x+ y+^ (4.5 %), Le^a+ b-^/Le^x+ y-^ (13.6 %) and Le^a- b-^/Le^x- y+^ (4.5 %) (Table 1).

**Table 1:** Distribution of ABO types, secretor status (FUT2), and Lewis antigens among 22 saliva donors

Saliva sample	Sex	Age	ABO types	FUT2	Lewis antigens
1	M	64	A	se-se-	Le^a+^Le^b-^/Le^x+^Le^y-^
2	F	8	A	se-se-	Le^a+^Le^b-^/Le^x+^Le^y-^
3	F	31	B	se-se-	Le^a+^Le^b-^/Le^x+^Le^y-^
4	F	63	A	Se + Se+	Le^a+^Le^b+^/Le^x-^Le^y+^
5	F	39	A	Se + se-	Le^a+^Le^b+^/Le^x-^Le^y+^
6	M	37	A	Se + se-	Le^a+^Le^b+^/Le^x-^Le^y+^
7	F	32	A	Se + se-	Le^a+^Le^b+^/Le^x-^Le^y+^
8	F	27	A	Se + se-	Le^a+^Le^b+^/Le^x-^Le^y+^
9	F	43	A	Se + se-	Le^a+^Le^b+^/Le^x-^Le^y+^
10	F	48	A	Se + se-	Le^a-^Le^b-^/Le^x-^Le^y-^
11	M	38	A	Se + se-	Le^a+^Le^b+^/Le^x-^Le^y+^
12	F	47	B	Se + Se+	Le^a+^Le^b+^/Le^x-^Le^y+^
13	M	58	B	Se + se-	Le^a+^Le^b+^/Le^x-^Le^y+^
14	M	31	B	Se + Se+	Le^a+^Le^b+^/Le^x-^Le^y+^
15	M	26	B	Se + se-	Le^a+^Le^b+^/Le^x-^Le^y+^
16	M	34	O	Se + se-	Le^a+^Le^b+^/Le^x-^Le^y+^
17	F	28	O	Se + se-	Le^a+^Le^b+^/Le^x-^Le^y+^
18	M	49	O	Se + se-	Le^a+^Le^b+^/Le^x+^Le^y+^
19	F	47	O	Se + se-	Le^a+^Le^b+^/Le^x-^Le^y+^
20	M	30	O	Se + se-	Le^a+^Le^b+^/Le^x-^Le^y+^
21	F	27	O	Se + Se+	Le^a+^Le^b+^/Le^x-^Le^y+^
22	F	27	O	Se + Se+	Le^a+^Le^b+^/Le^x-^Le^y+^

### NoV VLP binding to porcine gastric mucin (PGM)

**Fig. 1:**
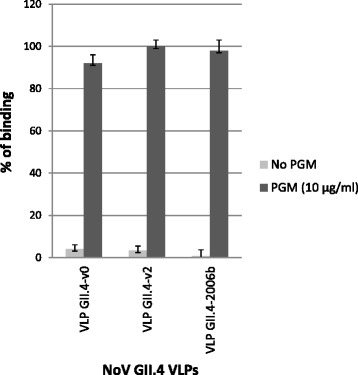
Binding of NoV VLPs (GII.4-1999 (v0) and GII.4-2004 (v2) and GII.4-2006b) to porcine gastric mucin (PGM). Microtiter plates coated with PGM (10 μg/ml) or without PGM (PBS) were incubated with the different VLPs at 2 μg/ml for 1 h and processed for detection of bound VLPs by ELISA, using an anti-NoV VLP rabbit polyclonal antiserum (pAb). Binding is expressed as percentages referred to the highest OD 450 nm value obtained in duplicate assays. Error bars indicate standard deviations

All VLPs (GII.4-v0, GII.4-v2 and GII.4-2006b) bound to PGM. Binding was detected with the anti-NoV rabbit antiserum (pAb) and no binding differences were observed among the three variants tested. VLP binding to PGM was specific, since no reactivity was detected in the control wells (Fig. 1).

### NoV VLP binding to saliva: different strains show distinct binding patterns determined by HBGAs

**Fig. 2:**
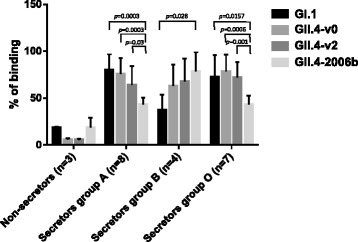
Mean values of NoV VLP binding to saliva samples expressed as percentages. NoV VLPs (G1.1, GII.4-v0, GII.4-v2 and GII.4-2006b) were assayed for their ability to bind to saliva samples from 22 volunteers including non-secretors (*n* = 3) and secretors with blood group A (*n* = 8), B (*n* = 4) and O (*n* = 7). Error bars indicate standard deviations. *P* values indicating significant differences between NoV VLP binding are shown

Binding assays to saliva were performed to analyse the NoV VLP binding patterns using saliva from non-secretors (*n* = 3) and secretors (*n* = 19) that were also divided in 3 groups depending on their blood group types (A, B and O). Four recombinant NoV VLP variants representing genogroups I and II were analysed: GI.1 (Norwalk virus), GII.4-1999 (v0), GII.4-2004 (v2) and GII.4-2006b. Although a limited number of samples were tested, a clear distinction between secretors and non-secretors was observed, since the saliva from non-secretors bound very inefficiently any of the VLPs (Fig. 2). The results of the binding assay to the saliva from secretor positive donors indicated that human NoV genogroups I and II exhibit distinct HBGA binding patterns (Fig. 2). GI.1 bound more efficiently to saliva from blood type A and O donors than to type B saliva samples, GII.4-v0 and v2 variants recognized samples from all blood groups (A, B and O) and GII.4-2006b VLPs recognized more efficiently saliva samples from blood group B donors.

### NoV VLP binding to saliva is inhibited by pre-incubation with anti-Lewis and anti-NoV mAbs

**Fig. 3:**
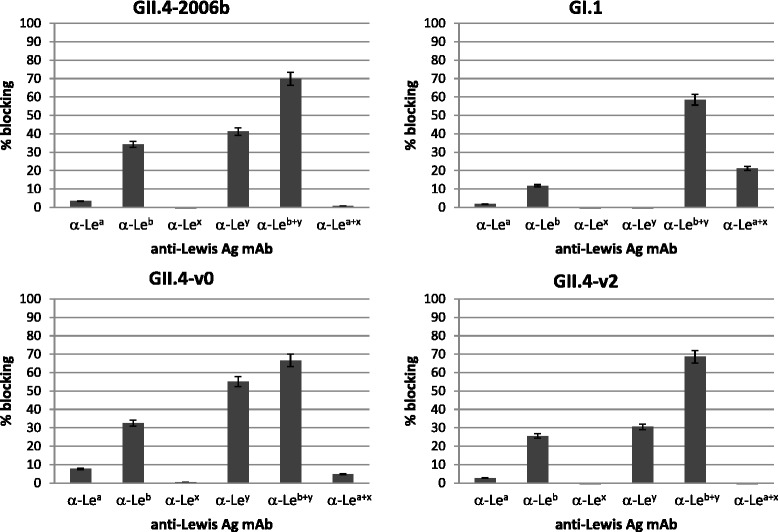
Blockade of the NoV VLP binding to saliva from a secretor (SeSe) individual by monoclonal antibodies (mAbs) against human Lewis antigens. Microtiter plates coated with saliva were preincubated with mAbs against Le^a^, Le^b^, Le^x^, Le^y^ or with a combination of Le^a+x^ or Le^b+y^ antigens and then the VLPs (GII.4-v0 and v2, GII.4-2006b and GI.1) were added. Graphs show the means of duplicate tests. Specific blocking of VLP binding was determined by comparing the percentage of reduction of the OD values in wells with mAbs compared to wells without mAbs. Error bars indicate standard deviations of the means

Incubation of secretor (SeSe, O-type, Le^a+^Le^b+^/Le^x-^Le^y+^) saliva-coated wells with anti-Le^y^ or anti-Le^b^ resulted in blocking of the binding of all GII.4 VLP variants to saliva, with an additive effect observed when anti-Le^b^ and anti Le^y^ were used in combination, resulting in 60 to 75 % reduction in the binding of the different NoV VLP variants when compared to untreated wells (Fig. 3). Anti-Le^a^ or anti-Le^x^ alone or in combination did not strongly block the binding to secretor saliva samples.

**Fig. 4:**
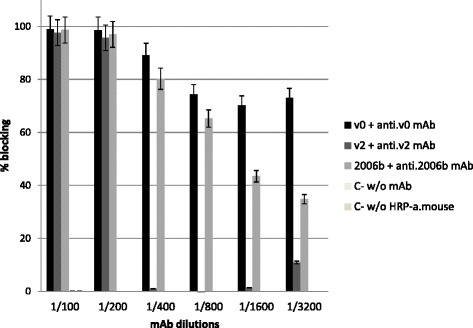
Blockade with anti-NoV mAbs of VLP binding to saliva samples. Microtiter plate wells were coated with saliva from a secretor individual at 1:500. Each VLP variant was preincubated with its homologous mAb before being added to the plate at 37 °C and detected with HRP-conjugated to anti-mouse IgG. All dilutions of anti-NoV antibodies reduced the binding with the homologous VLP variant compared to the controls in a dose-dependent manner. Error bars represent standard deviation

Anti-NoV mAbs tested prevented the binding of their homologous NoV VLPs to the saliva from an O blood group individual. Complete binding inhibition was seen when the mAbs were used up to a 1:200 dilution, whereas the blocking activity of anti-v2 mAb sharply drop out at 1:400 dilution and the anti-v0 and anti-2006b mAbs blocked the binding till higher dilutions (Fig. 4).

### NoV VLP binding to D-Caco-2 cells

**Fig. 5:**
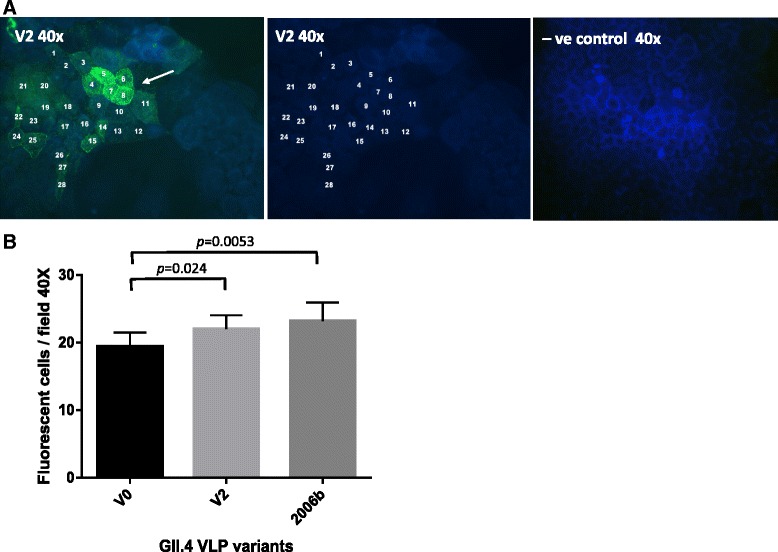
Fluorescence microscopy analysis of the attachment of the GII.4 2004 variant NoV VLPs (v2) to D-Caco-2 cells. **a** Representative immunofluorescence microscopy images of D-Caco-2 cells in the presence of 5 μg/ml of v2 VLPs (*left*). Binding of VLPs (shown a v2 strain) was performed by incubating D-Caco-2 cells with VLPs followed by detection using an anti-NoV polyclonal antibody as described under the ‘Methods’ section. The arrow indicates VLP binding and numbers mark off the cells bound by v2 VLPs. Negative controls correspond to D-Caco-2 cells incubated without VLPs (*right*). The image on the middle shows the cell monolayer under DAPI filter. **b** Mean counts of nine microscopic fields (40X) with standard deviations of D-Caco-2 cells bound with NoV GII.4-v0, GII.4-v2 and GII.4-2006bVLPs (fluorescent cells/microscopic field). *P* values indicating significant differences are shown

VLPs of all three GII.4 variants bound to D-Caco-2 cells. Binding was not uniform across the entire cell preparation, and VLPs could be detected only on certain cells or clusters of the cell layer. As an example, fluorescence microscopy images showing the attachment of 2004 variant NoV VLPs (v2) to D-Caco-2 cells are shown in Fig. 5. No signals were detected in any of the negative controls without VLPs.

### Blockade of VLP binding to D-Caco-2 cells

**Fig. 6:**
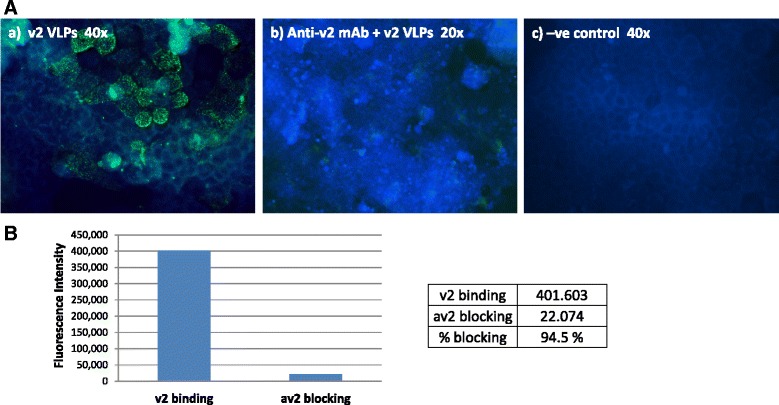
Blockade of binding of NoV VLPs to D-Caco-2 cells by anti-NoV mAbs. **a** (a) D-Caco-2 cells were incubated with GII.4-2004 variant (v2) VLPs. (b) Same concentration of VLPs was pre-incubated with the homologous variant-specific mAb (at 1:100 dilution of the antibody) and added to D-Caco-2 cells; (c) negative control incubated with PBS. **b** Measurement using the ImageJ software program of the fluorescence intensity revealing NoV VLPs (v2) bound to D-Caco-2 cells and its blockade by anti-v2.mAb

Anti-NoV mAbs used in this study strongly blocked the attachment of NoV VLPs to the surface of D-Caco-2 cells, and with the same strain-specificity as that observed in the salivary assays. Consistent with the ELISA results, a 74 % binding reduction was observed when VLPs were pre-incubated with the av2.mAb prior to adding to the cells (Fig. 6a). The blocking activity of the av0.mAb was of 82 %, whereas anti-2006b mAb also blocked the VLP binding (54.5 % reduction) (Table 2). When the VLPs were incubated with different PGM concentrations, blockade of NoV VLPs binding to the D-Caco-2 cells occurred in a dose-dependent manner. At a concentration of 10 μg/ml, PGM completely abolished the ability of NoV VLPs to bind to D-Caco-2 cells. PGM at a concentration of 1 μg/ml caused a 50 % reduction of VLPs bound to the cells, and no blocking effect was seen at a concentration of 0.1 μg/ml (data not shown). Figure 6b shows the blockade by anti-v2.mAb of VLPs binding to D-Caco-2 cells. Fluorescence intensity was measured using the ImageJ software program, which demonstrated a 94.5 % blocking activity.

**Table 2:** VLP binding blockade by NoV GII.4 variant-specific mAbs

	NoV GII.4 variant VLP binding to D-Caco-2 cells^a^		
mAb	GII.4 1999 (v2)	GII.4-2004 (v0)	GII.4-2006b
none	23 ± 5	17 ± 2	22 ± 3
av2 mAb	6 ± 2 (74 %)	-	-
av0 mAb	-	3 ± 1 (82 %)	-
3C3G3 mAb	-	-	10 ± 2 (54.5 %)

^a^ Mean values ± SD of fluorescent cell counts in ten microscopic fields (40X). The blocking of VLP binding to D-Caco-2 cells was performed with mAbs by preincubating the different VLPs for 1 h with the test antibodies diluted to 1:100 in PBS or with PBS alone for the negative control. Blocking percentages are indicated between parentheses

### Analysis of D-Caco-2 cells HBGA diversity reveals high expression of H-type 2, Le^y^ and Le^x^ antigens

**Fig. 7:**
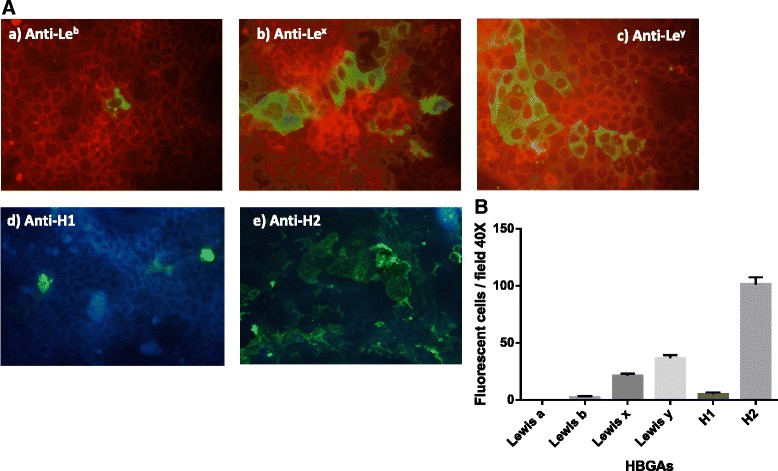
Heterogeneous expression of HBGAs on D-Caco-2 cell surface. **a** Immunofluorescence assay on D-Caco-2 cells fixed with methanol. Lewis and H antigens were detected by anti-Le^a^ (images not shown), Le^b^, Le^x^, Le^y^, H1 and H2 antibodies, followed by FITC-labeled anti–mouse IgG. Cells were counterstained with Evans blue (red fluorescence). HBGA expression in D-Caco-2 cells was evaluated by counting immunofluorescent cells in 10 microscopic fields (40X). (a and d) Weak expression of both Le^b^ and H1 antigen (score 1+), respectively. (b and c) Strong expression of Le^x^ and Le^y^ (score 2+). (e) Very strong H2 antigen expression on D-Caco-2 cell surface (score 3+). **b** Mean counts of 10 microscopic fields (40X) with standard deviations of D-Caco-2 cells immunostained with anti-Le^a^, Le^b^, Le^x^, Le^y^, H1 and H2 antibodies (fluorescent cells/microscopic field). All differences were statistically significant (*p* < 0.01)

To determine if anti-Lewis (anti-Le^a^, Le^b^, Le^x^ and Le^y^) and/or anti-type H (H1 and H2) antigen mAbs could influence and block the VLP binding to D-Caco-2 cells, the expression and distribution of these antigens on the surface of the D-Caco-2 cells were investigated. The majority of D-Caco-2 cells expressed H-type 2 antigen on their surface. High expression of Le^y^ and Le^x^ antigens was also observed, whereas Le^b^ and H-type 1 antigens were sparsely expressed (Fig. 7).

### NoV VLPs bind to D-Caco-2 cells independently of Le^y^ and H-type 2 antigen expression

**Fig. 8:**
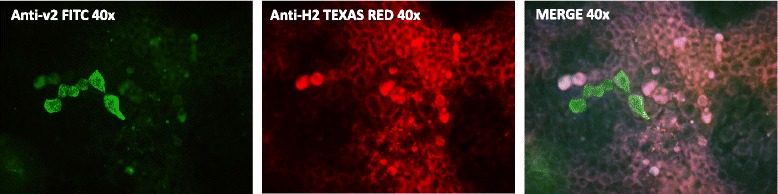
NoV VLPs bind to D-Caco-2 cells independently of H type-2 antigen expression. GII.4-2004 (v2) VLPs were added to D-Caco-2 cells after adding anti-H2 antigen. After 1 h incubation at 37 °C with an anti-H2 mAb, the cells were fixed and incubated with secondary FITC-conjugated anti-rabbit IgG (to detect VLP binding) or Texas red-conjugated anti-mouse IgG (to detect anti-H2) antibodies. No co-localization of H2 antigen expression and VLP binding was observed

Preincubation of D-Caco-2 cells with anti-Lewis Le^y^ and anti-H-type 2 mAbs did not result in any relevant blocking of GII.4 binding to the D-Caco-2 cells. Blockade by these antibodies was tested because of the high expression of Le^y^ and H-type 2 antigens on the surface of D-Caco-2 cells. Furthermore, HBGA expression did not co-localise with the VLP binding, as it is shown as an example in Fig. 8 with GII.4-2004 (v2) VLPs and the H-type 2 antigen.

## Discussion

HBGAs are neutral carbohydrates which are present on the surface of erythrocytes and mucosal epithelial cells, or as free oligosaccharides in milk, saliva and intestinal fluid of secretor individuals [27]. They are synthesized by sequential enzymatic transfer of single carbohydrate residues to specific precursor carbohydrate substrates, with very high genetic polymorphism [28]. Previous studies suggested that HBGAs are likely to play an important role as cell receptors for NoV attachment [29, 30], although the molecular mechanism leading to cell entry and infection are to date largely unknown.

NoV binding assays using salivary EIAs have in recent years become the technique of choice for the study of the binding specificities of NoVs to HBGAs, since saliva samples containing different HBGA expression profiles are easy to collect, and the methods are relatively simple and fast to perform. Moreover, assays using synthetic HBGA have proven to be challenging in terms of reproducibility, but also troublesome due to the unavailability or difficulties in obtaining such reagents. In the absence of a reliable cell culture system or an appropriate animal model for human noroviruses, the NoV VLPs and HBGA binding assays have contributed significantly to our understanding of host susceptibility to NoV infection and have also allowed the mapping of regions responsible for the interaction between HBGAs and the P2 subdomain [10, 31, 32].

In this study we have identified the HBGA binding profiles of four different NoV VLPs representing genogroups I (GI.1) and II (GII.4-1999 (v0), GII.4-2004 (v2) and GII.4-2006b) using saliva samples from different individuals. Four different binding patterns based on the secretor status, blood type and Lewis phenotype have previously been described [9, 30]. The VLPs used in this study exhibited distinct HBGA binding patterns: GI.1 VLPs bound more efficiently to saliva type A and O than to saliva type B, GII.4-v0 and v2 variant VLPs bound to all types A, B and O, and GII.4-2006b VLPs bound most efficiently to saliva type B. These results are in agreement with those reported by Uusi-Kerttula et al. [21], who analysed the binding of different NoV genotypes and GII.4 variants to salivary and synthetic HBGAs.

While many studies have compared results of binding assays to saliva and to synthetic HBGAs, the present study also compares NoV VLP binding patterns to saliva and to the human intestinal cell line Caco-2. Polarized or D-Caco-2 cells are also known to be a useful tool for the study of NoV binding, as both native NoVs and VLPs bind and penetrate the cell surface despite their inability to lead to productive infection [22, 33–35]. We have used Caco-2 cells to understand host-virus interactions because it has been demonstrated that they can differentiate in culture into cells with high homology to mature enterocytes in the intestinal epithelium [36, 37]. Moreover, they express HBGAs, like Lewis or H-type antigens [17, 22, 38].

Our experiments with D-Caco-2 cells clearly demonstrated that VLPs bound to certain subpopulations of cells, and anti-NoV antibodies abolished binding to these cells. We performed the VLP binding assays to D-Caco-2 cells at 37 °C, likely to represent physiological conditions more accurately. Previous work has been performed at 4 °C to prevent internalization which was reported to occur in 5-7 % of the cells when incubating at 37 °C [35]. However, similar internalisation has also been reported even when incubation is performed at 4 °C [22].

Using HBGA-specific mAbs to localise HBGAs on the surface of D-Caco-2 cells showed widespread expression of H2 and Le^y^, and more diffuse or clustered expression of H1, Le^b^ and Le^x^. Our observations are similar to those previously reported by Murakami et al. [22], and contrast with others reporting a high type H1 antigen expression [38]. However, Caco-2 are a heterogeneous cell population and the expression of morphological and functional characteristics depend on the degree of differentiation, which may explain the different results observed in relation to HBGA antigen expression, making it difficult to compare results obtained by different laboratories. Blocking HBGA on the surface of D-Caco-2 cells with specific mAbs did not affect NoV VLP binding, furthermore, no co-localisation of HBGA and NoV VLPs was observed by immunofluorescence. These results therefore suggest that binding to Caco-2 cells could be mediated by receptors other than HBGAs, in agreement with a recent report by Murakami et al. [22]. Some viruses require interactions with more than one cellular surface molecule to initiate their replication cycle, for example bovine herpesvirus [39], and adenovirus [40]. Furthermore, some NoV strains, such as GII.4-2004, fail to bind efficiently to any HBGA suggesting they may bind other carbohydrates [41, 42], e.g. negatively charged sugars, similar to feline calicivirus (FCV) [43] or murine norovirus (MNV) [44]. Specifically, FCV attach to a α 2, 6-linked sialic acid residue, but uses the junctional adhesion molecule 1 (JAM-1) for internalization into host cells [45]. Murine norovirus, in contrast, binds to a ganglioside GD1a present on the surface of murine macrophages [44]. In support of these hypotheses, some studies have demonstrated binding of GII noroviruses to negatively charged heparan sulfate [34], sialylated Lewis^x^ carbohydrate [46], H-type 3 antigen [21] and gangliosides [47]. Furthermore, studies have shown that HBGA association is not sufficient to overcome the failure to propagate these viruses in vitro, as exemplified by the resistance to infections of cell lines expressing HBGAs [23, 48]. It must be acknowledged that the expression and potential role of other HBGAs such as H3 and H4 was not investigated here, and this requires further study.

We propose that the D-Caco-2 cell binding assays may be more suitable for studying in vitro human NoV attachment and virus-host interactions than the salivary assays, which may only be considering the HBGA-NoV interactions, and are also likely to provide a more reliable surrogate for neutralising assays.

## Conclusions

The interaction of NoV GII.4 genotype variants VLPs with human secretor positive saliva samples and with D-Caco-2 cells is mediated by different attaching receptors. Although their binding to saliva occurs according to distinct HBGA binding patterns and can be blocked by antibodies against Lewis antigens, their attachment to D-Caco-2 cells may be dependent on other receptors, which still need further investigation.

## Methods

### Recombinant NoV VLP expression

Copy DNA derived from capsid genes of NoV strains GI.1 and GII.4-Den Haag_2006b were cloned and expressed by the Bac-to-Bac baculovirus expression system (Invitrogen, Paisley, UK), and recombinant NoV VLPs were produced in Sf9 insect cells following described procedures [49]. VLPs were purified from the cellular fraction lysed with Triton X-100 and centrifuged through a 40 % (w/v) sucrose cushion. VLPs of strains GII.4-1999 (v0) and GII.4-2004 (v2) were also expressed by recombinant baculoviruses as previously described [13]. The three GII.4 variants were chosen by their reported distinct binding properties [15, 50]. The different NoV VLP preparations were examined by electron microscopy (EM) after negative staining with 2 % phosphotungstic acid for purity, morphology and integrity appraisal.

### Saliva samples

Saliva samples were collected from 22 healthy adult volunteers, none of whom had taken any medication at the time or around the time of sample collection. To minimize the effects of the circadian rhythm, saliva samples were consistently collected in the morning hours (between 8 and 10 a.m.). Also, participants were instructed not to smoke, eat, drink, or brush their teeth in the 2 h before saliva collection. Freshly collected saliva samples were centrifuged at 10,000 × g for 5 min to remove particulate material, host and microbial cells and boiled at 100 °C to inactivate antibodies. Supernatants were collected, divided into several aliquots and stored at -80 °C until their use. Blood ABO typing of the saliva donors was performed by hemagglutination assays (ALBAclone® Monoclonal ABO Antisera, Alpha Laboratories, Eastleigh, England). Lewis antigens were phenotyped in the saliva samples by ELISA with monoclonal antibodies against Le^a^, Le^b^, Le^x^ and Le^y^ (Covance, Dedham, MA, USA). Briefly, plates were coated with saliva samples at 1:1000 dilution in carbonate/bicarbonate buffer (pH 9.6) at 4 °C overnight. After blocking with PBS containing 3 % (w/v) bovine seroalbumin (PBS-BSA), plates were incubated with the different anti-Lewis antigen monoclonal antibodies (anti-Le^a^ BG-5, Le^b^ BG-6, Le^x^ BG-7 or Le^y^ BG-8, Covance) at 1:100 during 1 h at 37 °C and detected by a secondary mouse antibody mix (anti-IgG, anti-IgM and anti-IgA) (Sigma) conjugated to horseradish peroxidase (HRP). After each step, plates were washed with PBS containing 0.05 % of Tween-20 (PBS-T). The reaction was developed by the addition of OPD Fast (Sigma, Dorset, England) and stopped after 10 min incubation with 3 M H_2_SO_4_. Absorbance was measured at 492 nm in a microplate reader (Multiskan FC, Thermo Scientific, Vantaa, Finland).

The secretor (FUT2+) and non-secretor (FUT2-) status was investigated by genotyping the FUT2 gene. Genomic DNA was extracted from saliva samples using the Qiagen QIAamp DNA Mini Kit. Genotyping for *FUT2* was performed by PCR-RFLP as described previously [51].

### Monoclonal (mAb) and polyclonal (pAb) antibody production

BALB/c mice were immunized by intraperitoneal (IP) inoculation of three doses of GII.4-2006b VLPs with Freund’s adjuvant at 15-day intervals. Splenocytes were fused with Sp2/0-Ag14 mieloma cells as described previously [52] and hybridomas were screened by ELISA using the same NoV VLP genotype as the antigen. An anti-GII.4-2006b was obtained (3C3G3) and used along the present study. The anti-v0.mAb and anti-v2.mAb were previously obtained as described [13]. A polyclonal antiserum (pAb) against NoV VLPs was obtained at the Public Health England (PHE, London) by immunizing rabbits with a mixture of VLPs (GII.4-v0, GII.4-v2 and GII.3).

### Enzyme-linked immunoassays (EIAs)

#### VLP binding to porcine gastric mucin (PGM)

Microtiter plates were coated with PGM at 10 μg/ml in carbonate-bicarbonate buffer (pH 9.6) at 37 °C overnight. Plates were washed three times with PBS-T (PBS containing 0.05 % Tween-20), blocked with PBS containing 1 % skimmed milk and incubated 30 min at 37 °C. VLPs were added at 2 μg/ml in PBS and incubated 1 h at 37 °C. After three washes, the anti-VLPs rabbit polyclonal antiserum (pAb) was added at 1:1000 dilution. Binding was detected with HRP-conjugated anti-rabbit IgG at 1:10,000 dilution (Bioss, Woburn, MA). The reaction was developed by the addition of TMB (3,3’,5,5’-tetramethyl-benzidine, Sigma) and stopped after 10 min incubation with 4 N H_2_SO_4_. Absorbance was measured at 450 nm in a microplate reader Multiskan FC (Thermo Scientific). Negative and blank controls were included in all assays.

### Blocking assays

The same EIA protocol used to measure NoV VLP binding to HBGAs in saliva was followed as described [9], adding an incubation step with the different mAbs before the addition of the VLPs, and/or preincubating the VLPs in the presence of the NoV-specific antibodies. Monoclonal antibodies against Lewis antigens were added to the plate at a dilution of 1:10. NoV VLPs (GI.1, GII.4-2006b, GII.4-v0 and GII.4-v2) were added at 2-10 μg/ml depending of the VLP variant, and their detection was performed with the anti-NoV rabbit polyclonal serum followed by a secondary HRP-conjugated anti-rabbit IgG antibody diluted at 1:10,000 (Bioss, Woburn, MA). The ability of anti-NoV mAbs to block the binding of VLPs to saliva was tested using serial 2-fold dilutions of each mAb from 1:100 until 1:3200. The VLPs (2 μg/ml in PBS) were pre-incubated with the assayed antibodies, or with PBS in the negative control, for 1 h at 37 °C. The mixtures were then added to the saliva-coated plates and incubated for 1 h at 37 °C. The binding of VLPs was determined by incubation with HRP-conjugated anti rabbit or anti-mouse IgG antibodies (1:2000 dilution), as appropriate, followed by the peroxidase substrate TMB step.

Blocking of binding to PGM was determined using the assay format described, but coating with PGM (5 μg/ml) the microtiter plates. Blocking of VLP binding by the tested antibodies was determined by comparing the OD values obtained in wells in duplicate containing potential blocking reagents against the control wells (without the blocking steps).

### Caco-2 cell binding assays

#### Caco-2 cell culture

Caco-2 cells were grown in Dulbecco’s modified Eagle’s minimum essential medium (DMEM) (Invitrogen) supplemented with 4.5 % glucose, 10 % (v/v) fetal bovine serum (Invitrogen), non-essential amino acids, 100 I.U./ml penicillin, 100 mg/ml streptomycin, 2 mM glutamine and 1 mM sodium pyruvate, at 37 °C under a humidified atmosphere with 5 % CO_2_. Binding medium (BM), used when the cells were incubated with the VLPs, contained all of the above except fetal bovine serum.

### Cell binding assays

Caco-2 cell binding assays were perfomed as previously described [17] with modifications. Caco-2 cells were seeded at 2 × 10^5^ cells/ml on culture slides (BD Falcon, Bedford, MA). Cells were cultured for 9-10 days to allow cell differentiation (D-Caco-2). D-Caco-2 cells were washed twice with PBS and incubated with VLPs (5 μg/ml) in 200 μl BM at 37 °C for 1 h, under a humidified atmosphere. Negative controls consisted of cells incubated without VLPs. After 3 washes with PBS, cells were fixed with methanol for 15 min at room temperature and then blocked with PBS containing 1 % skimmed milk (PBS-milk 1 %) for 30 min at 37 °C. Fixed cells were subsequently incubated at 37 °C for 1 h with polyclonal rabbit IgG anti-NoV VLPs (PHE) antibody at 1:4000 dilution in PBS. After washing the cells with PBS-0.05 % Tween-20, they were incubated with a goat FITC-conjugated anti-rabbit IgG antibody (Abcam, Cambridge, UK) at 1:1000 dilution for 1 h at 37 °C. Finally, slides were mounted with 10 % glycerol in saline and preparations were observed under a Nikon Eclipse 80i fluorescence microscope equipped with a digital camera and the Hamamatsu camera controller C4742-95 (Hamamatsu City, Japan).

### Expression of HBGAs

Immunofluorescence assays were performed as described above in order to analyse the expression of Lewis antigens, using anti-Le^a^ BG-5, Le^b^ BG-6, Le^x^ BG-7 and Le^y^ BG-3 monoclonal antibodies (Covance) and anti-H antigens (mAb to blood group H1(O) and anti-blood group H2 antigen, Abcam). Anti-Lewis antigen mAbs were used diluted to 1:100 and anti-H antigens mAbs to 1:200. For visualization, a secondary antibody FITC-conjugated anti-mouse IgG antibody (Santa Cruz Biotechnology) diluted 1:400 was used. HBGA expression in D-Caco-2 cells was evaluated by counting immunofluorescent cells in 10 microscopic fields (40X). A semiquantitative four stage scoring system was applied with the following ranges: negative (-); < 50 fluorescent cells (1+); 51-199 fluorescent cells (2+), and >200 fluorescent cells (3+).

### VLP-cell binding blocking assays

To assess the ability of anti-HBGA-specific antibodies to block the binding of VLPs to D-Caco-2 cells, cell chambers were incubated with anti-Le^y^ or anti-H-type 2 mAbs (as described for the HBGA expression assay) prior to incubation with the NoV GII.4-v2 VLPs. In order to study the ability of the anti-NoV antibodies to block the binding of the NoV VLPs to D-Caco-2 cells, VLPs were incubated with their homologous antibody using the same conditions as in the salivary blocking assays, but with BM as the diluent. Blocking of VLPs binding to D-Caco-2 cells by PGM was performed by incubating the GII.4-v2 VLPs for 1 h at 37 °C with PGM at a concentration of 0, 10 and 50 μg/ml. The rest of the procedure was described in the binding assays section. Control cells were incubated with buffer instead of mAbs/VLP, and VLPs in the absence of mAbs or PGM were used as positive binding controls. Staining specificity was assessed in negative controls by (1) omission of the VLP step and (2) replacement of primary antibodies with 3 % BSA/PBS. Immunofluorescence was assessed on a quantitative scale by calculating the number of positive cells on each well chamber in 10 microscopic fields (40X). Fluorescence intensity of the VLP-cell binding and blocking assays was measured using the Java-based image processing ImageJ 1.49 program [53].

### Statistical analysis

The nonparametric Mann-Whitney test was applied to determine the presence or absence of significant differences. In all cases *p*-values below 0.05 were considered statistically significant. The analyses were performed by using the IBM SPSS software vs. 22.0 (IBM Corp.).

### Ethics statement

Appropriate informed written consent was obtained from all four volunteers and the saliva samples were analysed anonymously. The study protocol and consent forms were approved by the Human Research Ethics Committee of the University of Valencia. The animal studies were evaluated and approved by the Animal Experimentation and Welfare Ethics Committee of the University of Valencia. JB possesses the accreditation by the Conselleria de Agricultura, Generalitat Valenciana, to design and perform experiments with laboratory animals.
